# Notes

**Published:** 1899-08-12

**Authors:** 


### NOTES.

The Duke of Portland has intimated his acceptance of the presidentship of the Westminster General Dispensary, Gerrard Street, Soho, vacant by the death of the Duke of Northumberland.

A new convalescent home has been opened on the East Cliff at Folkestone. The home, which will be named after the founder, Mrs. Means, is well situated, and contains 24 cubicles for patients and 12 beds in separate bedrooms. The cost is estimated at £750.

A definite arrangement as to the disposal of the Manchester Royal Infirmary site has nearly been completed. Three thousand yards are to be preserved for the infirmary to erect an out-patient department and receiving house. The remainder of the land will then be purchased by the Corporation for the sum of £350,000. The Corporation propose to pay £250,000 in cash, and to collect £50,000 from the public, and the municipality expect to pay the remaining £50,000 in yearly amounts of £2,500.

A quarterly court of the governors of the Hospital for Consumption and Diseases of the Chest, Brompton, was held on the 3rd inst., Mr. T. P. Beckwith in the chair. The report, read by the Secretary (Mr. W. H. Theobald), stated that the funds for the proposed "Country Branch and Convalescent Home" were coming in very slowly, and help for this desirable object was urgently needed. The following bequests were announced: Mr. William H. May, £1,000, duty free; Miss Emily Adelaide Dow, share of residue reversionary.

